# Role of Hypoxia and the Adenosine System in Immune Evasion and Prognosis of Patients with Brain Metastases of Melanoma: A Multiplex Whole Slide Immunofluorescence Study

**DOI:** 10.3390/cancers12123753

**Published:** 2020-12-13

**Authors:** Arnulf Mayer, Maximilian Haist, Carmen Loquai, Stephan Grabbe, Matthias Rapp, Wilfried Roth, Peter Vaupel, Heinz Schmidberger

**Affiliations:** 1 Department of Radiation Oncology, University Medical Center of the Johannes Gutenberg University, 55131 Mainz, Germany; Maximilian.Haist@unimedizin-mainz.de (M.H.); matthias.rapp@unimedizin-mainz.de (M.R.); vaupel@uni-mainz.de (P.V.); heinz.schmidberger@unimedizin-mainz.de (H.S.); 2 Department of Dermatology, University Medical Center of the Johannes Gutenberg University, 55131 Mainz, Germany; carmen.loquai@unimedizin-mainz.de (C.L.); stephan.grabbe@unimedizin-mainz.de (S.G.); 3 Institute of Pathology, University Medical Center of the Johannes Gutenberg University, 55131 Mainz, Germany; wilfried.roth@unimedizin-mainz.de

**Keywords:** hypoxia, adenosine, tumor microenvironment, immunosuppression, multiplex immunohistochemistry, spatial statistics, immune checkpoint inhibitor, ipilimumab, radiotherapy

## Abstract

**Simple Summary:**

The introduction of immune-checkpoint inhibitors improved the therapeutic landscape for patients with advanced malignant melanoma. However, many patients, including patients with melanoma brain metastases, do not derive benefit from immune-checkpoint blockade. Hence, biomarkers are needed to identify potential mechanisms of resistance and optimize patient selection. This study aimed to explore the role of hypoxia-mediated immunosuppression within the tumor microenvironment of patients with metastatic melanoma using multiplex immunofluorescence. We analyzed the prognostic relevance of the hypoxia surrogate marker GLUT-1, the adenosine-synthesizing ectoenzymes CD73/CD39, and the infiltration by CD8 positive lymphocytes, and evaluated their spatial interaction within the tumor microenvironment (TME). Finally, we outlined the role of the melanoma immune phenotype for the patient’s prognosis and discussed the importance of tumor hypoxia and the adenosine system in shaping the tumor immune phenotype.

**Abstract:**

Following the introduction of immune checkpoint inhibitors, a substantial prolongation of the overall survival has been achieved for many patients with multiple brain metastases from melanoma. However, heterogeneity between individual tumor responses is incompletely understood. In order to determine the impact of the individual tumor phenotype on the prognosis of melanoma patients, we examined surgical sections from 33 patients who were treated with radiotherapy (whole-brain radiotherapy, WBRT, stereotactic radiotherapy, STX, or both) and Ipilimumab. We analyzed multiplex staining of the hypoxia marker GLUT-1, the adenosine (ADO)-associated enzymes CD73 and CD39, and CD8, a marker of cytotoxic T lymphocytes (CTL) on a single-cell basis using QuPath. Additionally, the MOSAIC interaction analysis algorithm was used to explore the hypothesis that CTL systematically avoid GLUT-1^high^ tumor areas. Our results revealed, that a strong GLUT-1 expression, low numbers of CTL, or exclusion of CTL from the tumor were correlated with significant prognostic detriment. Hypoxic tumors overall have smaller amounts of CTL, and spatial analysis revealed a repellent effect of hypoxia on CTL. In contrast to in vitro studies, specific upregulation of ADO-related enzymes CD73 and CD39 in GLUT-1^high^ tumor regions was never observed. In this study, we could show direct in vivo evidence for hypoxia-mediated immunosuppression in melanoma. Moreover, this study suggests a significant prognostic relevance of the tumor immune phenotype, the strength of CD8 infiltration in the tumor, and the expression of hypoxia marker GLUT-1 on melanoma cells. Last, our results suggest a temporal stability of the microenvironment-mediated immunosuppressive phenotype in melanoma.

## 1. Introduction

Before the introduction of targeted therapies and, in particular, immune checkpoint inhibitors (ICI), patients with brain metastases of malignant melanoma had a very limited survival prognosis of roughly five months [1,2,3]. Melanoma was the first tumor in which an ICI, the anti-CTLA-4 monoclonal antibody (mAb) Ipilimumab (IPI), showed breakthrough success [4,5]. The efficacy of therapy with ICI has been documented both by an increase in the median survival and by the occurrence of long-term survivors [6,7,8]. Despite this success of ICI in patients with metastatic melanoma, most studies have been conducted excluding patients with active melanoma brain metastases (MBM). Since approximately 40% of patients with malignant melanoma at a locoregional or metastatic stage will develop MBM, an effective treatment approach for these patients is needed [2,3]. Radiotherapy is effective against MBM [1] and may promote anti-tumor immune responses via induction of immunogenic cell death (ICD).

While combined radiation and immunotherapy improves overall survival (OS) in MBM patients, [7,9,10,11] many still do not derive a benefit [12,13]. Hence, biomarkers are needed to identify potential resistance mechanisms, optimize patient selection, and sequencing of therapies [14].

Several findings from experimental studies suggested that at least some of the resistance mechanisms that prevent successful immunotherapy in melanoma are not caused by the intrinsic anergy of immune cells. Instead, they seem to have their origin in the tumor microenvironment. Based on a publication from 1968, this hypothesis is referred to as Hellström’s paradox [15]. E.g., antigen-specific T cell responses can be reduced in tumor tissue, but effective in the healthy tissues in the same animals [16]. Explicitly referring to melanoma, a publication from 1986 [17] demonstrated that tumor tissue infiltrating T cells recognize tumor-associated antigens, but do not develop productive anti-tumor activity in vivo. Only after isolation of these cells, in vitro cultivation and subsequent stimulation with IL-2, cytotoxic activity was observed.

Hypoxia is of major pathophysiological relevance in the microenvironment of melanoma, as is the case for many other solid tumors [18,19,20]. As early as 1997, Lartigau and colleagues were able to show by direct, invasive measurements with needle electrodes that metastases of melanoma contain significantly reduced oxygen partial pressures compared to the corresponding healthy tissues [21]. Oxygen deficiency can mediate immunosuppressive effects via several mechanisms [20]. E.g., it has been shown that the competition for the energy-rich substrate glucose in the hypoxic tumor microenvironment alone can weaken the effectiveness of immune responses [22]. It appears, however, that the immunosuppressive effects of the hypoxic tumor microenvironment are more distinct.

Under hypoxic conditions, the nucleoside adenosine (ADO) accumulates in the extracellular compartment, mainly as a consequence of the catabolism of ATP. Based on a considerable number of experimental studies, ADO is considered to be a “metabolic immune checkpoint” [23]. In addition to the production of ADO as a metabolite of ATP degradation, there are secondary pathways through which extracellular ADO can be generated [23,24]. The main path, according to current knowledge, implies two membrane-bound enzymes that catalyze the sequential hydrolysis of extracellular ATP to adenosine [23]. During this process, ATP is degraded by the ectonucleotidase CD39 (ENTPD1) from ATP to AMP, which is then consecutively metabolized to ADO by the ecto-5’ nucleotidase CD73 (NT5E). ADO then interacts with specific G-protein coupled receptors, in particular with the ADO receptor A2A (A2AR), and thus exerts both paracrine and autocrine effects [25].

ADO has a plethora of effects on the immune system which are beyond the scope of this communication [26]. In the setting of our patient tissue samples, we wanted to explore whether the presence of ADO could be a causative factor for a reduced infiltration of tumor tissue by CD8-positive cytotoxic T-lymphocytes (CTL). [20,27] Since ADO cannot be detected in fixed tissue specimens, we chose to visualize the two ADO-producing ectoenzymes, which have been shown to be expressed in malignant melanoma [28,29] and which are associated with hypoxia [30].

We used multiplex immunofluorescence on patient tissue sections in combination with the digitization of whole tumor slices. This technological platform enables single cell-based quantitative analyses of up to several million cell elements in a spatial context. In addition to the antigens mentioned above needed for the identification of the respective cell population or marker expression (CD8, CD73, CD39), we used glucose transporter (GLUT)-1 as an endogenous surrogate marker of hypoxia.

For a total of 33 of 42 patients of a previously published clinical study [6], one or more tumor samples were available for analysis (a total collection of 69 specimens). This clinical dataset with extended follow-up allowed us to correlate tissue parameters with the overall survival of the patients. All patients received a combination of radiotherapy (see Methods for details) at the time of diagnosis of cerebral metastases and were also put on immunotherapy with IPI in close temporal proximity.

We speculated that CD8-positive CTL might show a systematic avoidance behavior towards tumor areas with an intense GLUT-1 expression (in the following referred to as GLUT-1^high^), in spatial analyses. A potential correlation of elevated CD73 and CD39 expression with microregional hypoxia (which has been assessed by the expression of hypoxia surrogate marker GLUT-1 on tumor cells) was evaluated as a risk factor for survival of these patients since an immunologically less active tumor microenvironment (TME) might diminish the response to immunotherapy [31] as well as radiotherapy (RT).

## 2. Results

### 2.1. Separating Tumor, Stroma, and CTL in Preparation for the Analysis

Identification of the neoplastic melanocyte compartment as a region of interest (ROI) formed the basis of all further analyses. Definitive identification of neoplastic cells in multiplex fluorescence specimens was based upon commonly used pathological criteria (e.g., a high nucleus to cytoplasm ratio) of melanoma cells and cross-validation by comparison with the corresponding H&E stained tumor sections. As described in the Methods section, after recognition of tumor cells by QuPath’s cell segmentation algorithm, samples were first separated into tumor and stroma compartments and then subclassified for antigen expression patterns. This preparatory step enabled us to quantify fractions of the tumor and stromal cells for each tumor section and to perform all further quantitative analyses separately for the two compartments.

### 2.2. The Occurrence of Hypoxic Areas in the Examined Tumor Sections

Due to the agreement of the staining patterns of the exogenous hypoxia marker pimonidazole and antibodies against the antigen carbonic anhydrase IX (CAIX) shown in other tumor entities [32], the latter antigen would have been our first choice as an endogenous hypoxia marker. However, according to the publicly available results of immunostains in the human protein atlas (proteinatlas.org), 12 different tumor samples of melanoma detected with three different antibodies against CAIX show no or at best very weak staining for this marker. Preliminary tests in our laboratory also confirmed this finding (Supplementary Materials Figure S1A). We, therefore, chose GLUT-1 as an endogenous hypoxia marker, which was expressed in 17 to 82% (median: 42%) of tumor cells in the tumors investigated (Table 1). We found expression of GLUT-1 in melanoma cells to be widespread and intense. It followed a pattern that reflects the diffusion properties of oxygen in the tissue, i.e., GLUT-1 expression intensity increased with increasing distance of the positive cells relative to the nearest blood vessel. In some specimens, the transition from GLUT-1 negative to positive cells was relatively abrupt. In contrast, in other cases, the transition was smoother, so that a degree of basal expression of the marker in melanoma appears to be present in a cell type-dependent manner.

### 2.3. Expression of the ADO-Generating Enzymes CD73 and CD39 and Associated Findings

**Figure 1:**
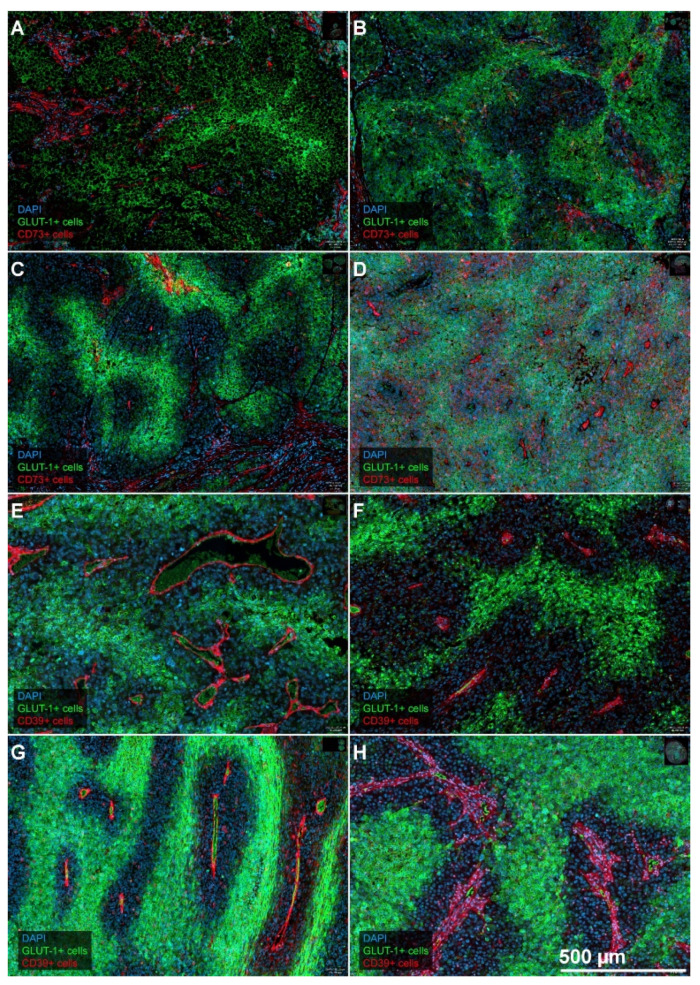
Representative examples for the combined expression patterns of GLUT-1 and CD73, and CD39. Examples from eight different patients (**A**): LN-metastasis; (**B**): LN-metastasis; (**C**): LN-metastasis; (**D**): Lung-metastasis; (**E**): Brain-metastasis; (**F**): Skin-metastasis; (**G**): LN-metastasis; (**H**): Skin-metastasis) showing the combination of GLUT-1 expression and CD73, panels A to D, or the combination of GLUT-1 and CD39, panels E to H. Both CD73 and CD39 are mainly expressed in the stroma and stromal microvessels. Expression in tumor cells is sometimes present as well, e.g., for CD73 in panel (**D**). GLUT-1 shows a “hypoxia-related” expression pattern since the expression is most pronounced at the greatest distances from adjacent intratumoral microvessels. In some tumors, the transition is quite abrupt (e.g., **B**,**C**,**F**,**G**,**H**), while it can be more gradual in others (**A**,**D**,**E**). Magnification, 10 ×. Scale bar in **H** applies to all panels of the figure.

Even though the expression of GLUT-1 thus met our pathophysiological expectations, this was not the case for CD73 and CD39. On the one hand, we found a weak positivity for CD73 on melanoma tumor cells (i.e., <14%) in the majority of patients (*n* = 21/33 cases) (Table 1). On the other hand, the pattern of a more extensive expression (fraction of CD73 positive tumor cells >14%; n = 12/33) of CD73 never showed the expected specific co-localization with GLUT-1^high^ tumor cell areas. These findings were similar for CD39. Indeed, in most specimens, both CD73 and CD39 were mainly located in the stroma, and here predominantly in the tumor microvasculature. Association with microvessels was visually evident due to the morphology of the positive structures but also confirmed after image registration with specimens stained for the vascular reference marker CD34 (Figure S2A). Figure 1 shows the expression pattern of GLUT-1 in relation to CD73 (panels A–D), or CD39 (panels 2E–H). The relationship to diffusion-limited oxygen availability, as made evident by the co-staining with the “vascular markers” CD73 and CD39 was present in all specimens in our cohort, but occasionally was not as pronounced as in the examples given in Figure 1.

### 2.4. Density and Distribution of CTL

For most of the tissues examined, we found a low density of CTL (median: 4.0%) in the whole-slide tumor sections. CTL were often already visually arranged in a compartmentalized fashion. Results show that CD8+ CTL regularly exhibit a pronounced clustering in the stroma and infiltrate melanoma cell aggregates to varying degrees depending on the phenotype of tumor immunity present in the whole-slide tumor (Figure S3A). Similar to the known pattern of an “excluded infiltrate” (see introduction), the main bulk of CTL was retained in the stromal septae, and only a tiny part of these cells had migrated into the tumor cell compartment. Conversely, staining for CD45, available in 19 of 33 cases, clearly showed that hematopoietic cells can penetrate between the tumor cells, which is consistent with what has been described for CTL (see discussion). We observed that CTL were significantly more prevalent in non-CNS-metastases compared to biopsies taken from CNS-metastases (mean CTL in non-CNS metastases: 7.1% vs. 1.2% in MBM, Mann–Whitney-test: *p* = 0.0004). This difference is consistent with the position of the CNS as an immune sanctuary site. Owing to the small number of CNS-specimens, these results should be interpreted with great caution.

### 2.5. Hypoxia-Induced Immune Evasion in Spatial and Statistical Analysis

**Figure 2:**
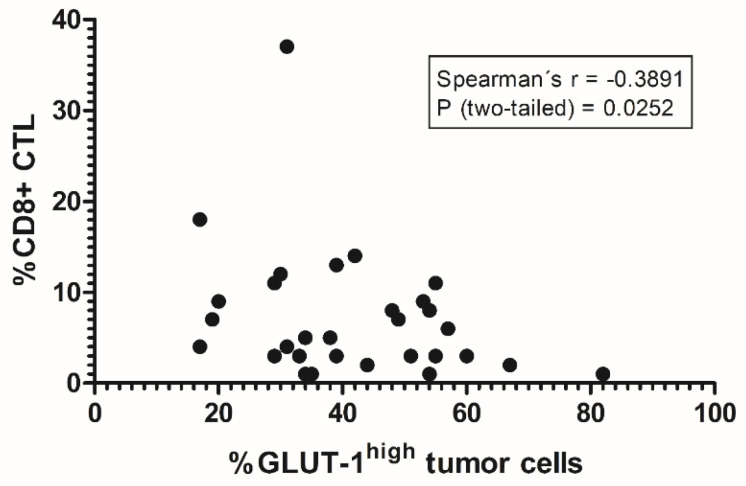
Inverse correlation between the extent of GLUT-1^high^ expression on tumor cells and cytotoxic T-lymphocytes (CTL) infiltration. On the level of the entire whole-slide images, the amount of GLUT-1^high^ tumor cells and the strength of the tumor infiltration by CD8+ CTL show a significant negative correlation (Spearman’s r: −0,39, *p* = 0.0252). This highlights the observation of systematic avoidance of presumably hypoxic, GLUT-1^high^ tumor cell areas by CTL. The tumor samples included in this analysis were derived from the following tissues: Brain (2), Skin of the Ear (1), Gastrointestinal tract (1), Lymph node (13), Lung (2), Myocardium (1), Mucosa of the palate (1), Rectum (1), and Skin (11). Statistical analysis was performed using a two-sided Spearman’s correlation analysis.

On the level of the entire specimens, an inverse correlation between the total amount of CTL and the extent of tumor cells showing a strong GLUT-1 expression (referred to as GLUT-1^high^) was found (r = −0.39; *p* = 0.025, Figure 2). Starting from the hypothesis that hypoxic areas, in particular, are an obstacle to the migration of CTL, the distribution pattern of CTL relative to GLUT-1^high^ positive areas was further analyzed in micro-regional interaction analysis using methods of spatial statistics. In most cases, either negative (i.e., interaction strength <0; *n* = 28) or weakly positive (interaction strength <5: *n* = 2) values for the strength of the interaction between the two cell populations were found using MOSAIC IA. This finding suggests a statistically significant rejection between CTL and the GLUT-1^high^ tumor compartment, thus indicating at the idea of hypoxia-mediated immune suppression. However, we found no quantitative correlation between the interaction strength values and the density of infiltration by CTL. Spatial analysis in MOSAIC IA also revealed statistically significant clustering of CD8+ and CD73+ cells in stromal areas, confirming our visual observations from the whole-slide tumor sections. By contrast, we could find no significant quantitative evidence for the exclusion of CTL from CD73+ tumor cell areas in metastatic melanoma neither on the level of the entire specimen, nor in the microregional spatial interaction analysis. In particular, we could observe an exclusion of CTL from CD73^high^ tumor cell areas only in a small fraction of metastatic melanoma samples (*n* = 6/69).

### 2.6. Prognostic Significance of the Investigated Markers

After the dichotomization of the patient cohort based on the median of the percentage of GLUT-1^high^ tumor cells in the tumor compartment, a significantly better prognosis was found for patients with a smaller fraction of GLUT-1^high^ tumor cells, i.e., for patients with better-oxygenated tumors. Patients whose tumor contained a maximum of 42 % of GLUT-1^high^ melanoma cells showed a median survival of 11 months, while patients with higher percentages of GLUT-1^high^ tumor cells had a median survival of only three months (log-rank *p* = 0.00067). Furthermore, a higher percentage of CD8+ CTL was associated with a better prognosis. If this proportion was more than 4%, the median survival was again 11 months, whereas patients with a percentage of CTL of 4% or less once more showed a median survival of only three months (log-rank *p* = 0.007). Notably, this correlation became stronger when analyzing only whole-slide tumor sections from non-CNS metastases (*p* = 0.0003), suggesting that the strength of CD8 infiltration as a prognostic factor might be dependent on the characteristics of the initial tumor specimens.

Next to the baseline number of CD8+ CTL, recent studies demonstrated that the spatial distribution of tumor infiltrating lymphocytes (TIL), characterized by the concept of tumor immune phenotype, might also have a prognostic impact and determine the response to ICI. Following these observations, we could show that the expression patterns of CTL were indeed significantly associated with prognosis, even when only the three patterns described in the literature were distinguished in our cohort. Patients with a dense infiltrate of CTL, the so-called “pre-existing immunity,” had a substantially better prognosis with a median survival of 38 months compared to patients with an “excluded infiltrate” (log-rank *p* = 0.033) or even an “immunological desert” (*p* = 0.003). The latter two phenotypes, at a lower level, again differed and showed a median survival of six and only one month (log-rank *p* = 0.019), respectively. Notably, we could observe a marked inter-individual heterogeneity in the manifestation of the so-called “immune-excluded” phenotype with substantial variations in the baseline CD8+ CTL numbers (between 1 % and 13%), as well as the overall survival in this subcohort (overall survival between 1 and 53 months).

**Figure 3:**
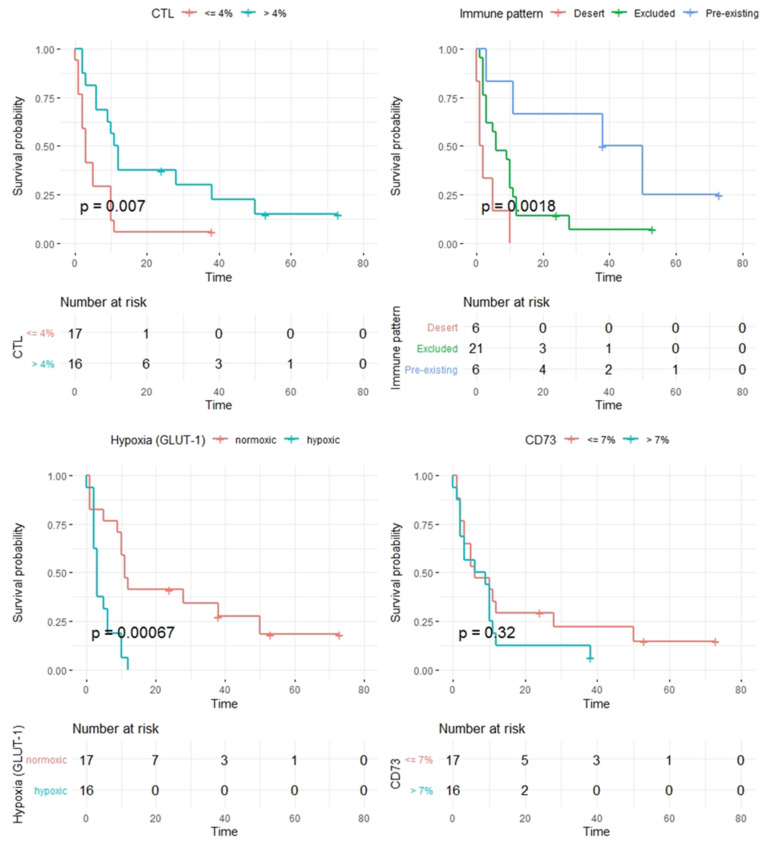
Kaplan–Meier plots for CD8+ CTL (upper left), immune phenotypes (upper right), GLUT-1^high^ positive tumor cells (bottom left), and CD73 positive tumor cells (bottom right).

Patients who exhibited the “excluded” phenotype were quite heterogeneous with regard to their clinical course. Therefore, in a separate analysis, we assessed the number of CD8+ CTL in the tumor surrounding stroma. Interestingly, we found a longer OS (10 vs. 3 months, *p* = 0.02) for patients with an excluded immune-infiltrate but high numbers of CD8+ CTL in the tumor surrounding stroma when compared to patients with a smaller number of CD8+ CTL in the stroma. By contrast, the amount of CD73+ or CD39+ tumor cells, as well as the quantitative amount of stroma in the examined tumors, showed no prognostic relevance at all. Kaplan–Meier plots showing the impact of CTL infiltration, immune pattern, GLUT-1 expression on tumor cells, and CD73 are shown in Figure 3.

## 3. Discussion

We obtained several findings that corroborate previous concepts of the pathophysiological role of the hypoxic melanoma microenvironment in immunosuppression. Our results put these elements into an in vivo context in human tumors which has not been shown previously. Our data also indicate that certain aspects of the concept of ADO-mediated immunosuppression need to be revisited and validated.

First, our results confirm the presence of extensive GLUT-1^high^ expressing tumor cell areas in melanomas and melanoma metastases in different tissues, which are correlated with an unfavorable prognosis. In particular, we found expression of GLUT-1 to be widespread, intense and organized in a pattern that reflects the diffusion geometries of oxygen in the tissue. In addition to the direct measurements of Lartigau [21], there are several studies on experimental tumors involving the exogenous hypoxia marker pimonidazole, which also show that melanomas are consistently hypoxic tumors. Concerns that such findings from xenograft tissue may not be transferable to metastases of human melanoma might be dispelled when, e.g., the distribution of pimonidazole in the landmark study of Lyng et al. [33] is compared with the distribution of GLUT-1 in our tumors. The patterns show a strong similarity.

In addition, Lyng’s work [33] has included measurements with polarographic needle electrodes on the same tumors, and these authors compared the results of both methods systematically. The patchy heterogeneous distribution of the hypoxia marker (compare Figure 1 in this report and Figure 4a in Lyng et al. [33]) corresponds to changing oxygen partial pressures in the electrode measurement tracks depending on the position of the needle within the tumor. In “Eppendorf studies,” the needle is gradually advanced deeper into the tissue during the measurement [34]. In numerous further investigations on experimental melanoma tumors, the same group of authors was able to show that the hypoxia represented by pimonidazole was primarily caused by a rarefication of tumor microvessels and increased diffusion distances (e.g., [35,36]). Based on the staining patterns of CD73, CD 39, and CD34 in relation to the GLUT-1^high^ positive areas, a similar pattern was recognizable in the human tumors investigated by us (Figure 1). This suggests that the intensity of GLUT-1 expression on melanoma cells might reflect the degree of tumor hypoxia. Our findings are consistent with previous results by Dura and coworkers [37]. These authors also describe increasing GLUT-1 expression with increasing distances from the stroma, which they refer to as “zonation”. However, contrary to us, they describe this pattern only in “some cases” of melanoma with Breslow thickness >4 mm. Interestingly, Dura et al. [37] found no expression of GLUT-1 in benign naevi, a finding which is compatible with prior data which show that benign tumors may not activate the hypoxia-inducible factor (HIF) system, even when they are hypoxic [38]. Findings consistent with the data of Dura et al. have been reported by Koch and coworkers [39]. Additionally, both studies [37,39], like the present study, describe a negative prognostic impact of a high GLUT-1 expression.

Second, GLUT-1^high^ (i.e., presumably hypoxic) tumors, in particular, showed the most pronounced exclusion of cytotoxic T lymphocytes from the tumor areas together with an overall low number of these cells. This reduced infiltration by CTL is also associated with an unfavorable prognosis, a fact that per se has also been presented in various prior publications. Seven of these studies have been summarized in the meta-analysis by Fu and colleagues [40]. Besides, hypoxia-mediated immunosuppression could already be demonstrated for head-neck cancer [41] and renal cell carcinoma [42]. Important and novel in our study, however, is the direct mechanistic relationship between an intense GLUT-1 expression on tumor cells and a decreased CTL infiltration in melanoma tumor samples, which we found in micro-regional interaction analyses and on the level of the entire patient cohort.

Our data are consistent with findings from previous studies in experimental tumors. The landmark study of Hatfield and coworkers [43] showed that hypoxic areas (Pimonidazole staining), present at baseline within the experimental tumors, contained significantly lower numbers of CD8+ CTL than normoxic areas. Respiratory hyperoxia effectively reduced the exposition of immune cells to hypoxic conditions and led to a significant increase in the density of CD8+ CTL. Our findings, for the first time, confirm the applicability of this pathophysiological model in human melanomas. We also presented a novel and robust algorithm for the assessment of the distribution of CTL in GLUT-1^high^ vs. GLUT-1^low^ tumor cell areas in this study. In addition, our spatial analysis showed that avoidance of GLUT-1^high^ tumor cell areas by CTL can be verified mathematically using the MOSAIC interaction analysis algorithm in the majority of the tumors investigated. However, the lack of standardized cut-off thresholds to dichotomize GLUT-1 positive tumor cell areas certainly remains a weakness of this methodology. Moreover, our conclusion that GLUT-1 might be a reliable biomarker for tumor hypoxia requires further validation.

Third, in our study, the clinical outcome for patients with MBM treated with a combination of RT and IPI was predicted from the analysis of tissue specimens which had in part been excised years before that stage in the course of their disease. This observation might indicate at a certain degree of temporal stability of the resistance mechanisms mediated by the tumor microenvironment, which is consistent with the observation of similar immune phenotypes in different tumor specimens of the same patient in our cohort (Figure S3A). In particular, the assessment of the tumor immune phenotype within melanoma lesions was found to be a robust prognostic factor. This is consistent with observations of Herbst and coworkers who demonstrated that the density and spatial distribution of CTL in melanoma lesions represented a reliable predictive indicator in response to PD-1 inhibition [27].

CTL infiltration in the tumor microenvironment alone, however, does not provide a comprehensive assessment of antitumoral immune activity [44]. The immune infiltrate rather represents a multifaceted component of the TME, which is determined by the interaction of tumor related factors, such as the tumor biology, and host factors, i.e., the ability of the patient’s immune system to respond effectively to tumors [44]. In the future, it will be possible to simultaneously visualize additional markers of immune cell activation and exhaustion [45,46] in approaches using higher multiplexing techniques, allowing for a more differentiated analysis (CTLA-4, LAG-3, CD62L, CCR7, or PD-1) [47]. These data could, e.g., allow a better differentiation between local effects of the tumor microenvironment and the overall immune status of the host.

However, the notion of the tumor microenvironment as a highly dynamic entity that is subject to a spontaneous variability of immune cell infiltration might not be entirely supported by our observations. In this regard, Tumeh et al. indicated that immunotherapy does not alter the overall spatial distribution pattern of immune cells, which defines the tumor immune phenotype, but rather modulates their baseline numbers within a given tumor specimen [48]. Therefore, these and other authors suggested that the tumor immune phenotype might exhibit, at least partially, temporal and spatial stability [49]. Interestingly, prior data also suggested initial priming and subsequent stability of the tumor microenvironment [50]. In a large number of experimental sarcomas in a rat model, we found that the initial priming of tumor-initiating cells in a hypoxic or normoxic milieu determines the nature of the subsequent tumor microenvironment to a remarkable degree. Tumors arising from cells cultivated in hypoxic ascites exhibited widespread microenvironmental hypoxia and activation of the hypoxia-inducible factor-system, while tumors derived from normoxic cell culture had a normoxic microenvironment. A systematic analysis of the temporal stability of the microenvironment of the melanoma and its metastases would, therefore, be of particular interest.

Regarding the role of the adenosine system in hypoxia-induced immunosuppression, our results have raised questions. Both CD73 and CD39 turned out to be predominantly expressed in the stroma, with a particular preference for intratumoral microvessels. The variable expression of these markers in the tumor cells of the melanoma itself showed (i) no specific correlation with the spatial distribution of the hypoxia-associated antigen GLUT-1 and (ii) no impact on patient prognosis. It is unlikely that this finding is the result of the technical shortcomings of our immunohistochemical approach. The biological plausibility of the strict co-localization of CD73 and CD39 in blood vessels is consistent with the model of biochemical activity of these proteins as tandem enzymes. Furthermore, a vessel-associated staining pattern can almost be ruled out as an artifact, since blood vessels are easily recognizable and unique structures which are never randomly generated by artificial processes. Besides, the expression of CD73 in blood vessels has already been described in other tumor entities, e.g., non-small cell lung cancer [51].

Regarding the expression of CD73 in melanoma, only the work of Monteiro et al. [28] has been published so far. This study mainly investigated the expression of CD73 in tumor cells and tumor-infiltrating monocytic cells. However, Figure 3D in Monteiro et al. [28] depicts a similar pattern of CD73 expression in stroma and blood vessels which we, using the identical primary antibody Clone D7F9A from Cell Signaling Technology, found in our study. Regarding the presence of CD73 in tumor cells, Monteiro’s publication [28] is consistent with our data. Bastid and colleagues [29] describe data regarding the expression of CD39 in melanomas derived from a commercial multi-tissue microarray. In their study, the staining of blood vessels by anti-CD39 was so typical that the authors refer to this component as their “internal positive control.” This is consistent with the predominating pattern of this antigen in vessels in our study. Kordaß et al. recently reviewed published data on the multitude of factors which are involved in the regulation of CD73 expression levels [52]. Hypoxia-independent pathways upregulating CD73 do exist and may not have attracted enough attention in the cancer immunotherapy community. Interestingly, one of the pathways capable of upregulating CD73 in the absence of hypoxia is the WNT/β-catenin signaling pathway, which has recently been found to mediate T-cell exclusion and resistance to ICI therapy in a mouse model of melanoma [53].

The findings of our study do not invalidate the concept of adenosine-mediated immunosuppression. As shown in the review by Vijayan et al. [23], this might partly be explained by other metabolic pathways by which extracellular adenosine can be generated. The rate-limiting factor in the biosynthesis of extracellular adenosine does not necessarily have to be the protein amount of the ectoenzymes CD73 and CD39. Under physiological conditions, extracellular ADO concentration might also be influenced by the rate of cellular uptake and metabolism by adenosine kinase (AK), an enzyme for which several pharmacological inhibitors have been generated, which increase extracellular ADO levels (reviewed in [54]).

In summary, the concept of hypoxia-mediated immunosuppression in the melanoma microenvironment via the downregulation of CD8+ CTL was strongly confirmed despite the small number of patients. A unique aspect of this study is the fact that the tissue specimens investigated had been excised long before the clinical challenge at the time, the occurrence of brain metastases, and their treatment with a combination of WBRT/SBRT, and IPI. This exciting finding may indicate that the stability of the melanoma microenvironment has not been sufficiently appreciated to date.

## 4. Patients and Methods

### 4.1. Patient Cohort

Patients were recruited from a previously published retrospective analysis of patients with MBM, which had been treated at our hospital between October 2010 and March 2015. The reader is referred to the respective publication for further details regarding the patient cohort [6]. The vast majority of samples came from non-CNS tissue (*n* = 63/69), which in many cases had partly been excised years before cerebral irradiation and immunotherapy (median: 8 months). All of these patients underwent hypofractionated whole-brain radiotherapy (WBRT), stereotactic radiotherapy (STX), or a combination of both techniques (Table 1). All received IPI, predominantly in a narrow time interval before or after irradiation. All patients received at least two applications, and a maximum of four applications of IPI (3 mg/kg) applied every three weeks in each case. One subgroup received IPI before radiotherapy, whereas the remaining patients received IPI after RT. Of the entire cohort of 41 patients, all 33 patients for whom one or more paraffin tissue blocks where available were included in the present study (69 tissue specimens from 33 patients in total). In the case of the eight patients for whom no material could be obtained, tissue had either been used entirely for diagnostic purposes, or tissue specimens had been sent out for analysis according to the requirements of other studies and were not available to us anymore. For the analysis of GLUT-1, CD73, and CD8, all 69 specimens were evaluated with the methods detailed below. Since this approach resulted in more than one tumor sample for some patients, a meaningful reduction of the quantitative data to a single score became necessary. Tumor specimens with the highest overall cell count were considered to represent the patient’s individual tumor best and were selected for correlation with the patients’ clinical data. Analyses of CD39 were selectively carried out only in the chosen specimens for CD73.

### 4.2. Immunofluorescence Staining

Immunofluorescence staining for the antigens GLUT-1, CD73, CD39, CD8, and, additionally, CD45, CD34, and Na^+^-K^+^-ATPase, was carried out according to a standard procedure as published previously [55]. In brief, after cutting 4 µm thick sections with high precision microtomes, specimens were incubated at 60 °C for one hour and deparaffinized in a descending alcohol series. Pretreatment for immunohistochemistry (IHC) was carried out using antigen-demasking buffers specific for the chosen antigen in each staining round (details are given in Table 1). Antibody incubation took place either overnight at 4 °C or for one hour at 30 °C, depending on the requirements for the specific antigen (Table 1). Primary antibody detection was carried out using appropriate secondary antibodies bound to an HRP–conjugated polymer (VECTOR ImmPRESS, Vectorlabs, Burlingame, CA, USA). Fluorescence tagging was finally carried out using a palette of fluorescyl-tyramide reagents (Table 2). Multiplexing of up to three antigens (in three series of stains) was achieved by quenching the peroxidase activity of the previous step using additional rounds of heat pretreatment. Buffers appropriate for the antigen, which was going to be investigated in the subsequent round of staining, were used in a modified version of the original protocol published by Toth and Mezey [56]. Additionally, all specimens were counterstained with DAPI at a concentration of 2.5 µg/mL for 5 min (Cat.-No. D1306, Thermo Fisher Scientific, Waltham, MA, USA). After rinsing with PBS, samples were covered with a coverslip using a fluorescence mounting medium (Cat.-No. S3023, Agilent, Santa Clara, CA, USA).

### 4.3. Quantitative Analyses

**Figure 4:**
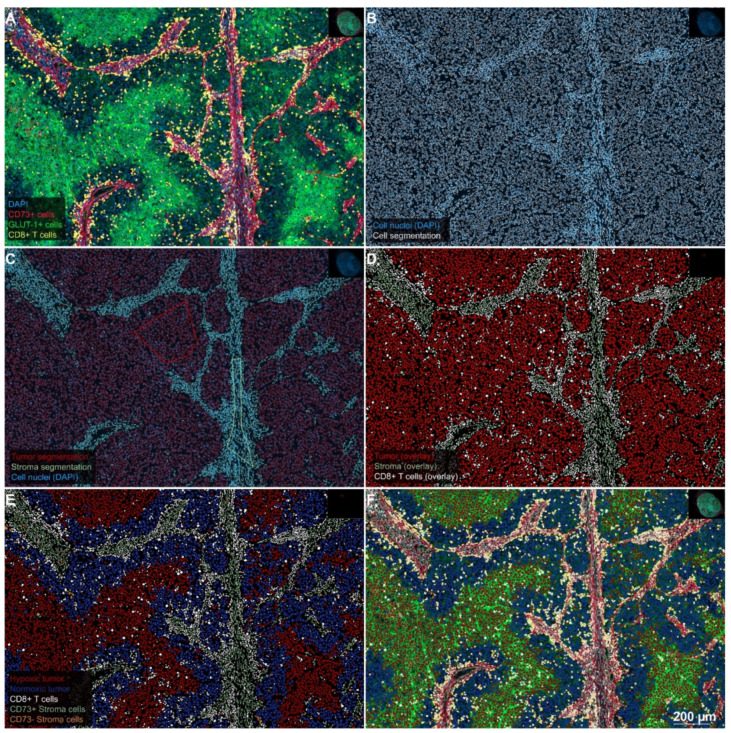
Stepwise procedure for the analysis of the number and spatial context of CD8-positive CTL in the hypoxic melanoma microenvironment. (**A**) Original scanned 4-plex-immunofluorescence image. A representative region of interest (5×) extracted from the whole-slide specimen of a patient’s lymph node metastasis demonstrates a heterogeneous staining pattern for GLUT-1 (FITC-channel, green), which reflects the diffusion-limited availability of oxygen in the melanoma microenvironment. The expression of CD73 (Alexa-647-channel, red) is largely limited to the stroma and the endothelium of tumor microvessels. CD8-positive CTL (Cy3-channel, yellow) show a strong clustering in the stroma, whereas the infiltration of CTL into GLUT-1^high^ tumor cell areas is sparse. Hence, the spatial distribution of CTL in this figure represents an example of an excluded infiltrate. DAPI counterstain (blue) was used for segmentation of cell nuclei. (**B**) Single-cell-based analyses were carried out using segmented nuclei (white border) as the starting point for cell detection in the open-source software QuPath. This approach enabled the systematic subclassification of all cell events. First, we subclassified cells as “tumor” or “stroma” using QuPath’s machine learning features and user-defined examples (**C**); tumor cells with a red border; stroma cells with an olive green border). Second, we further subclassified cells using intensity-thresholds in the relevant fluorescence channels in a sequential procedure, color-coding CTL with a white overlay based on the intensity in the Cy3 channel (**D**). Next, tumor cells (red overlay in **D**) were further subclassified into GLUT-1^high^ and GLUT-1^low^ (i.e., presumably hypoxic and normoxic) tumor cells with a red and blue overlay, respectively (**E**), based on thresholding in the FITC-channel. Finally, stroma cells (olive green overlay in D) were subclassified into CD73-positive and CD73-negative stroma cells, based on the intensity in the Alexa 647-channel (olive green and orange overlay, respectively as shown in **E**). Merging of the original 4-plex-IF-image (**A**) with the classification overlay (**E**), as shown in panel (**F**), illustrates the final result of this sequential approach. Despite a relatively dense immune infiltrate, this patient showed an overall survival of only ten months.

Single-cell-based analyses were carried out using the DAPI channel (blue) for the segmentation of cell nuclei in the open-source whole slide image analysis software QuPath (https://qupath.github.io/; [57]). Segmentation was followed by a stepwise procedure of further subclassification of cells (Figure 4). First, cells were classified as “tumor” or “stroma” using QuPath’s machine learning features and user-defined examples. Second, tumor and stromal cells were further subclassified using intensity thresholds in the relevant fluorescence channels. This subclassification comprised the identification of CD8-positive cells (i.e., CTL), hypoxic tumor cells, normoxic tumor cells, CD73/CD39-positive stroma, and CD73/CD39 negative stroma. To calculate the distance from each cell classified as (CD8-positive) CTL to the nearest hypoxic tumor cell, we used a custom script in QuPath. Quantitative data were correlated with clinical patient data. Tumors were also visually divided into three different phenotypes characterized by the pattern of CD8+ CTL infiltration [58]. The most common phenotype has shown to be the “excluded infiltrate” of CD8+ CTL (*n* = 21/33). In contrast, the other phenotypes did either show nearly no infiltration of immune cells in the tumor, known as “immunological desert” (*n* = 6/33) or an extreme infiltration of the entire tumor, referred to as “pre-existing immunity” (*n* = 6/33).

### 4.4. Spatial Analyses

Spatial analyses were carried out in the ImageJ plugin MOSAIC IA, which had been tailored to the specific needs of this study in prior collaborative work with the group of Professor I.F. Sbalzairini [59]. In particular, representative regions of interest chosen from the whole slide specimens in QuPath were transferred to ImageJ via QuPath’s ImageJ interface and subjected to MOSAIC’s spatial interaction analysis. Coordinates of cell centroids were used to estimate the direction and strength of the interaction between hypoxic tumor cells and CTL. MOSAIC returns data on the strength and direction of the interaction between the chosen cell populations (i.e., attracting or repellent) as well as a set of standard statistical parameters describing the distance distribution of the two cell populations relative to each other. Additionally, MOSAIC assesses the statistical significance of the interaction strength values using a Monte Carlo approach. Significant interactions were defined as having a *p*-value of < 0.05.

### 4.5. Statistical Testing

As a time-to-event endpoint, this retrospective cohort study used overall survival, which was estimated using the Kaplan–Meier product-limit method and log-rank statistics in R [60]. Moreover, the statistical analysis included the Mann–Whitney test and Spearman’s correlation analysis. These tests were performed for the quantitative analyses of CD8+ and hypoxic tumor cells to define significant differences in spatial CD8 CTL distribution (Mann–Whitney test) or correlations between the amount of CD8+ CTL and hypoxic tumor cells (two-sided Spearman’s correlation analysis).

## 5. Conclusions

In summary, we found that metastatic melanoma samples with a strong expression of the endogenous hypoxia marker GLUT-1 (“GLUT-1^high^”) contained a smaller amount of CTL. Moreover, spatial analyses revealed a microregionally repellent effect of GLUT-1^high^ tumor cells on CTL. These findings suggest an essential role of hypoxia-mediated immunosuppression in metastatic melanoma. A specific upregulation of ADO-related enzymes CD73 and CD39 in GLUT-1^high^ tumor regions, by contrast, was never observed. Finally, our results revealed that a strong GLUT-1 expression on tumor cells, low numbers of CTL, or exclusion of CTL from the tumor and an anergic immune phenotype were correlated with significant prognostic detriment.

## Figures and Tables

**Table 1:** Clinicopathological features of the investigated study cohort.

Clinicopathological Features	N (%)
Median age at initiation of RT (range)	59 (29–83)
Gender	
Female	10/33 (30.4%)
Male	23/33 (69.6%)
**Primary tumor**	
Melanoma type	
Cutaneous	28/33 (84.8%)
Ocular	1/33 (3.0%)
Mucosal	2/33 (6.1%)
Unknown primary site	2/33 (6.1%)
Breslow depth ^1^ (median)	2.2 mm
Clark-level ^1^ (median)	IV
BRAF mutated	14/33 (42.4%)
**Metastatic lesion**	
Biopsy sites (N = 69)	
Lymph node (LN)	24/69 (34.8%)
Skin and subcutaneous tissue	29/69 (42.0%)
Lung	3/69 (4.3%)
Central nervous system (CNS)	6/69 (8.7%)
Liver	1 (1.4%)
Gastrointestinal tract	3 (4.3%)
Other (Bone, Palate mucosae, myocard)	3 (4.3%)
Time between histological resection and IPI initiation (median)	8 months
RPA class (median)	2
**Quantitative data from immunofluorescence analysis**	
% CD8+ CTL median (range)	4 (1–37)
% GLUT-1^high^ tumor cells median (range)	42 (17–82)
% GLUT-1^low^ tumor cells median (range)	20 (2–58)
% CD73+ tumor cells median (range)	7 (0–72)
**Treatments**	
Previous treatments	
Treatment-naïve	3/33 (9.1%)
Previously treated	30/33 (90.9%)
Treatment received	
Chemotherapy	10/33 (30.3%)
Radiotherapy	
▪ STX alone ▪ WBRT alone ▪ WBRT and STX	14/33 (42.4%) 12/33 (36.3%) 7/33 (21.2%)
Ipilimumab	33/33 (100%)
Anti-PD1/anti-PD-L1 inhibitors	5/33 (15.1%)
BRAF ± MEK inhibitors	8/33 (24.2%)
IPI prior to RT	18/33 (54.5%)
Time between IPI initiation and RT (median)	1 month
**Follow-up**	
Median survival upon RT (range)	6 months (0–73 months)
Deceased	29/33 (87.8%)

^1^ Percentage based on the total number of patients with known Breslow’s depth (*n* = 25)/Clark-level (*n* = 19). Bold font indicates the subtitles in the table.

**Table 2:** Antibodies (AB), immunohistochemistry (IHC) protocol and resulting staining patterns.

Antigen	Primary AB (Cat.-No., Clone, Species, Dilution, Incubation)	Primary AB Supplier	Fluorochrome (Cat.-No.)	Fluorochrome Supplier	Staining Pattern
CD73/NT5E	13160, D7F9A, rabbit (mono), 1:50, for 1 h at 28−30 °C	Cell Signaling Technology, Danvers, MA (USA)	TSA Plus Cyanine 5 (NEL745001KT)	PerkinElmer, Waltham, MA, USA	See main text
GLUT-1	GTX62480, EPR3915, rabbit (mono), 1:500, overnight at 4 °C	GeneTex, Irvine, CA (USA)	Alexa Fluor 488 Tyramide (B40953)	Thermo Fisher Scientific, Waltham, MA, USA	Membranous
CD8	M7103, CD8/144B, mouse (mono), 1:50, for 1 h at 28−30°C	DAKO, Glostrup, Denmark A/S (DK)	TSA Plus Cyanine 3 (NEL744001KT)	PerkinElmer, Waltham, MA, USA	Membranous
Na^+^-K^+^- ATPase	ab76020, EP1845Y, rabbit (mono), 1:100, for 1 h at 28–30 °C	Abcam, Cambridge (UK)	Alexa Fluor 647 Tyramide (T20951)	Thermo Fisher Scientific, Waltham, MA, USA	Membranous
CD34	ab81289, EPR373Y, rabbit (mono), 1:100, overnight at 4 °C	Abcam, Cambridge (UK)	Alexa Fluor 488 Tyramide (B40953)	Thermo Fisher Scientific, Waltham, MA, USA	Membranous (endothelial cells)
CD45	M0701, 2B11+PD7/26, mouse (mono), 1:50, for 1 h at 28−30 °C	DAKO, Glostrup, Denmark A/S (DK)	TSA Plus Cyanine 3 (NEL744001KT)	PerkinElmer, Waltham, MA, USA	Membranous
CD39/ENTP1	NBP2-45447, OTI2B10, mouse (mono), 1:100, for 1 h at 28–30 °C	Novus Biologicals, Littleton, CO (USA)	TSA Plus Cyanine 5 (NEL745001KT)	PerkinElmer, Waltham, MA, USA	See main text

## Supplementary Materials

The following are available online at https://www.mdpi.com/2072-6694/12/12/3753/s1, Figure S1: Weak or absent expression of CA IX in melanomas; Figure S2. Co-localization of CD73 and CD34; Figure S3: Representative slices for the 3 tumor immune phenotypes observed in this study.

## Abbreviations

A2AR	A2A-receptor
ADO	Extracellular adenosine
CA-IX	Carbonic anhydrase IX
CCR	CC chemokine receptors
CNS	Central nervous system
CTL	Cytotoxic T-lymphocyte
CTLA-4	Cytotoxic-T-lymphocyte protein 4
DAPI	4′,6-diamidino-2-phenylindole
GLUT	Glucose transporter
H&E	Hematoxylin and eosin
HIF	Hypoxia-inducible factor
HRP	Horseradish peroxidase
ICD	Immunogenic cell death
ICI	Immune checkpoint inhibitor
IF	Immunofluorescence
IHC	Immunohistochemistry
IL	Interleukin
IPI	Ipilimumab
LAG3	Lymphocyte-activation gene 3
LN	Lymph node
mAb	Monoclonal antibody
MBM	Melanoma brain metastases
OS	Overall survival
PD-1	Programmed cell death protein 1
ROI	Region of interest
RT	Radiotherapy
STX	Stereotactic brain radiotherapy
TIL	Tumor-infiltrating lymphocytes
TME	Tumor microenvironment
WBRT	Whole-brain radiotherapy
