# Supplementary material for: Role of Hypoxia and the Adenosine System in Immune Evasion and Prognosis of Patients with Brain Metastases of Melanoma: A Multiplex Whole Slide Immunofluorescence Study
Source: Cancers (Basel). 2020 Dec 13;12(12):3753. doi: 10.3390/cancers12123753 (PMC7763902; doi:10.3390/cancers12123753)
Supplement: Supplementary file 1 [file cancers-12-03753-s001.pdf]

## Supplementary Materials

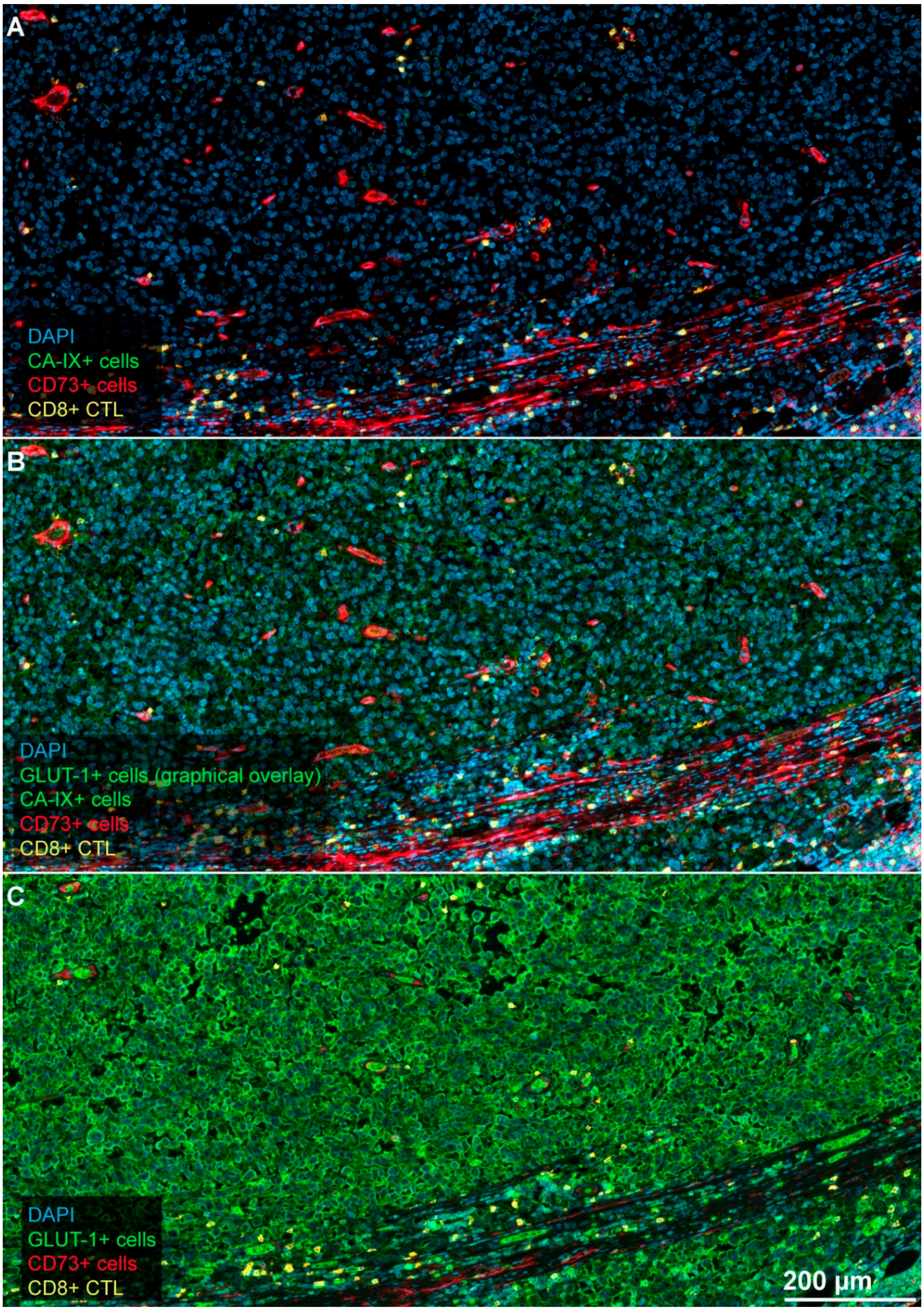


**Figure S1.** Weak or absent expression of CA IX in melanomas. Comparison of the staining patterns of carbonic anhydrase (CA)-IX and GLUT-1 using interactive image alignment in a lymph node metastasis of malignant melanoma. (**A**) CA-IX is shown in the green channel but is barely visible because the expression level of the antigen is shallow. (**C**) shows bright, positive staining for GLUT-1 in the same region of the same specimen, as illustrated by the graphical overlay after affine transformation shown in (**B**). Other channels: DAPI, blue; CD73, red; CD8, yellow. Magnification, 10x. Scale bar in (**C**) applies to all three panels.

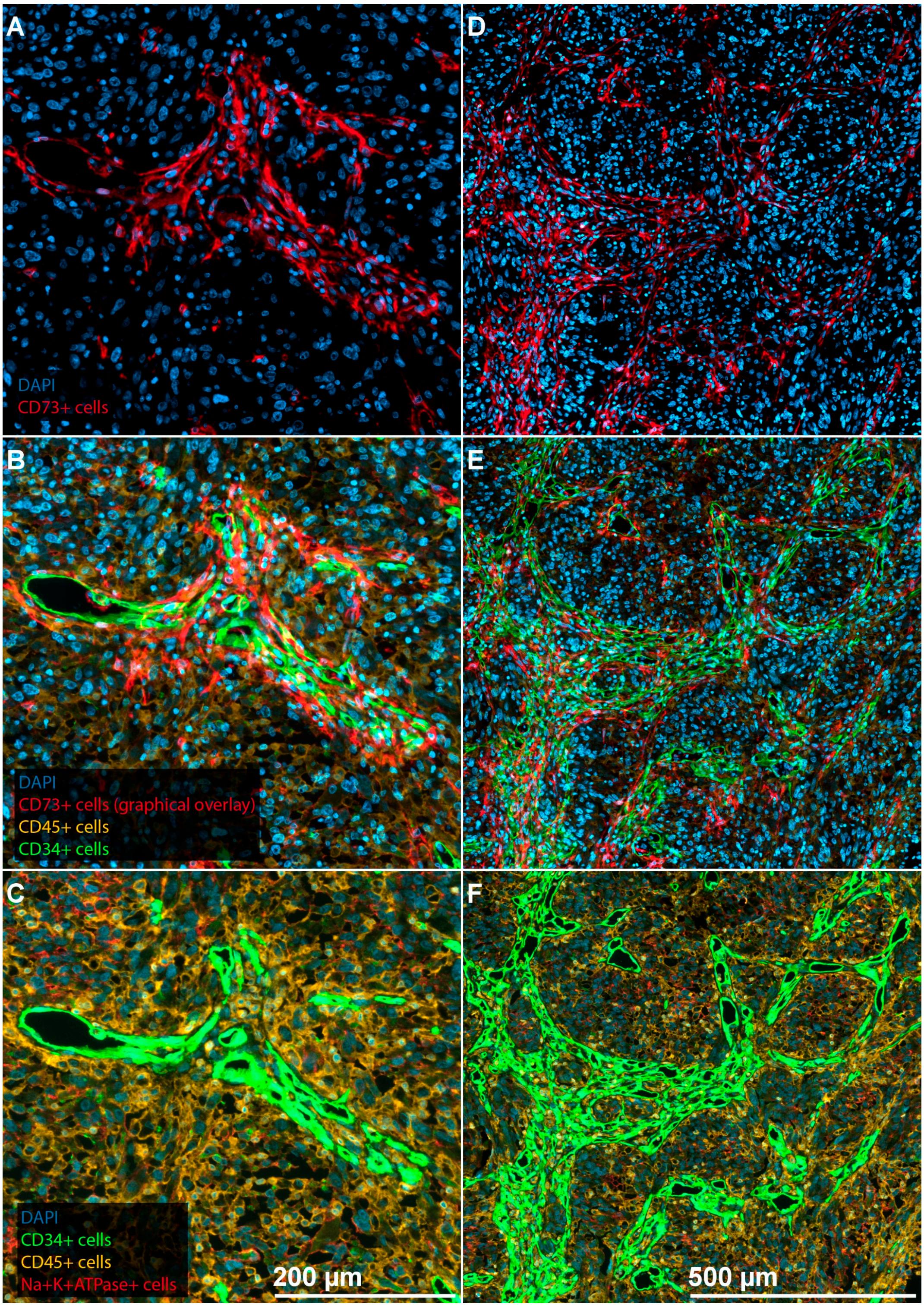


**Figure S2.** Co-localization of CD73 and CD34. Representative examples of CD73-positive tumor microvessels (red) and the corresponding CD34-staining (green) in a registered serial section are shown in 20x (panels **A** to **C**), and 10x (panels **D** to **F**) magnification. **A**, **D**, CD73 alone. **C**, **F**, CD34 (green) alone. **B**, **E**, overlay of both series. CD73- and CD34-positive cells exhibit an almost perfect match. While the first tumor section in the registration (target image) shows only the antigen of interest, i.e., CD73, and DAPI, the selection of channels was not possible in the second tumor section (source image containing CD34 staining). The orange signal in the CD34-section corresponds to CD45, while the weak red signal in **C** and **F** corresponds to Na-K-ATPase.

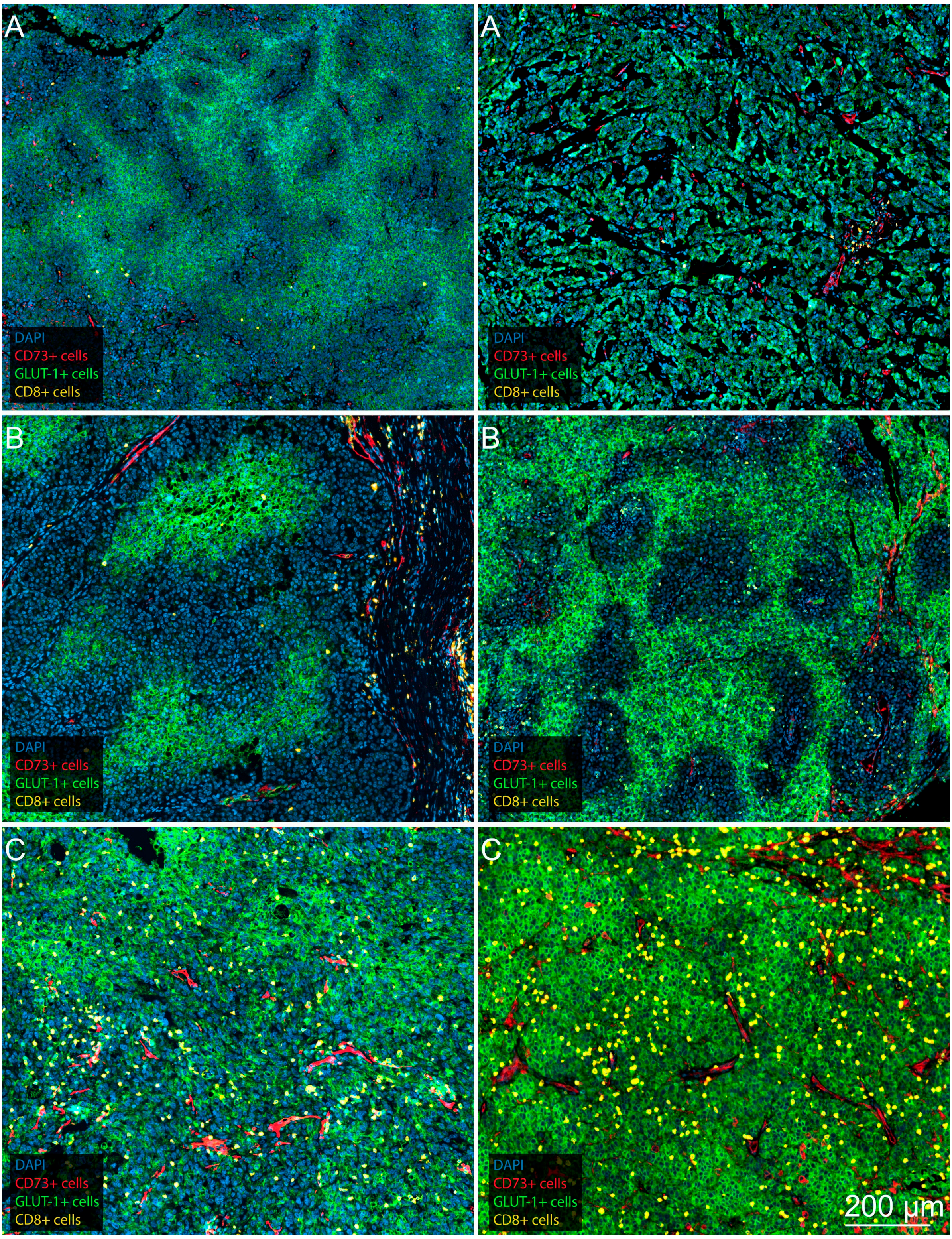


**Figure S3:** Representative slices for the 3 tumor immune phenotypes observed in this study. Each row represents two separate specimens from the same patient excised at different time points in the course of the disease. (**A**) Anergic tumor with minimal CTL infiltration (lung metastasis, left; skin metastasis, right). (**B**) Excluded immune infiltrate (both LN metastases). (**C**) Pre-existing immunity (myocarial metastasis, left; LN metastsis, right). Magnification, 5×. Scale bar in (**C**) applies to all panels.
